# Supplementary material for: Antioxidant and Cytotoxic Effects on Tumor Cells of Exopolysaccharides from Tetraselmis suecica (Kylin) Butcher Grown Under Autotrophic and Heterotrophic Conditions
Source: Mar Drugs. 2020 Oct 26;18(11):534. doi: 10.3390/md18110534 (PMC7693365; doi:10.3390/md18110534)
Supplement: Supplementary file 1 [file marinedrugs-18-00534-s001.pdf]

marine drugs

MDPI

Article

Antioxidant and antitumor activity of exopolysaccharides from *Tetraselmis suecica* grown under autotrophic and heterotrophic conditions

Geovanna Parra-Riofrío [1,2,*], Jorge García-Márquez [3], Virginia Casas-Arrojo [4], Eduardo Uribe-Tapia [2] and Roberto Teófilo Abdala-Díaz [4,*]

[1] Doctorado en Acuicultura. Programa Cooperativo Universidad de Chile, Universidad Católica del Norte, Pontificia Universidad Católica de Valparaíso, Chile
[2] Departamento de Acuicultura, Facultad de Ciencias del Mar, Universidad Católica del Norte, Larrondo 1281, Coquimbo, Chile; euribe@ucn.cl
[3] Department of Microbiology, Faculty of Sciences, University of Malaga, 29071, Malaga, Spain; j.garcia@uma.es
[4] Instituto de Biotecnología y Desarrollo Azul (IBYDA), Departamento de Ecología y Geología, Facultad de Ciencias, Universidad de Málaga, 29071, Málaga, Spain; virginiac@uma.es
* Correspondence: gbparrar@gmail.com (G.P.-R.); abdala@uma.es (R.T.A.-D.); Tel.: +56-966960044 (G.P.-R.); +34-952136652 (R.T.A.-D.)

Supplementary Material

Table S1. Pearson correlation between variables from heterotrophic culture of *T. suecica* (n=3)

	DPPH	Phenol	ABTS	Lipids	Chl a	Chl b	Carotens	Carbohydrates	Protein	TC	TN	C/N
**DPPH**	-	.9252**	.9133*	.9497**	-.8720*	-.8818*	-.8748*	.8928*	.8784*	.9274**	.9191*	-.6275
**Phenol**		-	.9956**	.9792**	-.9888**	-.9880**	-.9897**	.9783**	.9921**	.9936**	.9967**	-.7692
**ABTS**			-	.9686**	-.9870**	-.9849**	-.9872**	.9857**	.9929**	.9957**	.9995**	-.7741
**Lipids**				-	-.9654**	-.9581**	-.9662**	.9675**	.9665**	.9599**	.9752**	-.8222*
**Chl a**					-	.9961**	.9999**	-.9874**	-.9937**	-.9736**	-.9870**	.8258*
**Chl b**						-	.9967**	-.9843**	-.9847**	-.9761**	-.9833**	.7832
**Carotens**							-	-.9868**	-.9935**	-.9745**	-.9872**	.8215*
**Carbohydrates**								-	.9801**	.9687**	.9853**	-.8362*

**Protein**	-	.9829**	.9938**	-.8181*
**TC**		-	.9947**	-.7161
**TN**			-	-.7836
**C/N**				-

* *p < 0.05*; ** *p < 0.01*.

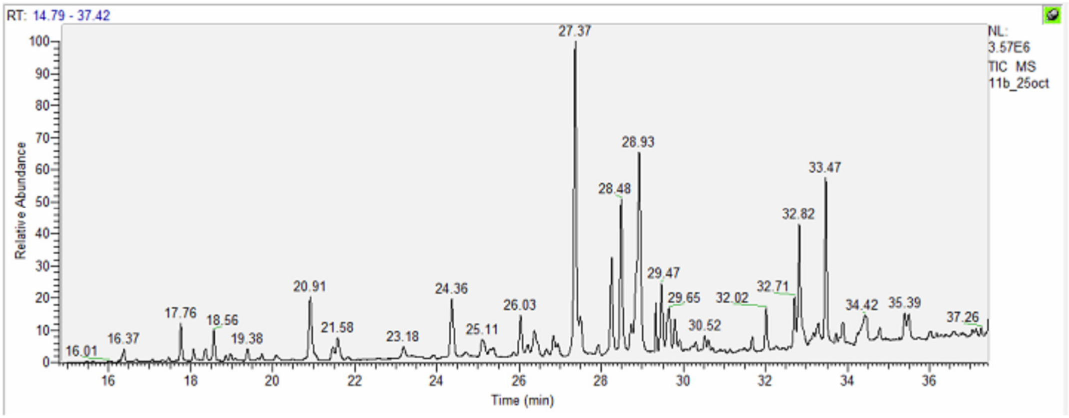

Figure S1. Gas chromatography-mass spectrometry (GC-MS) analysis of total EPS obtained from autotrophic culture of *T. suecica.*

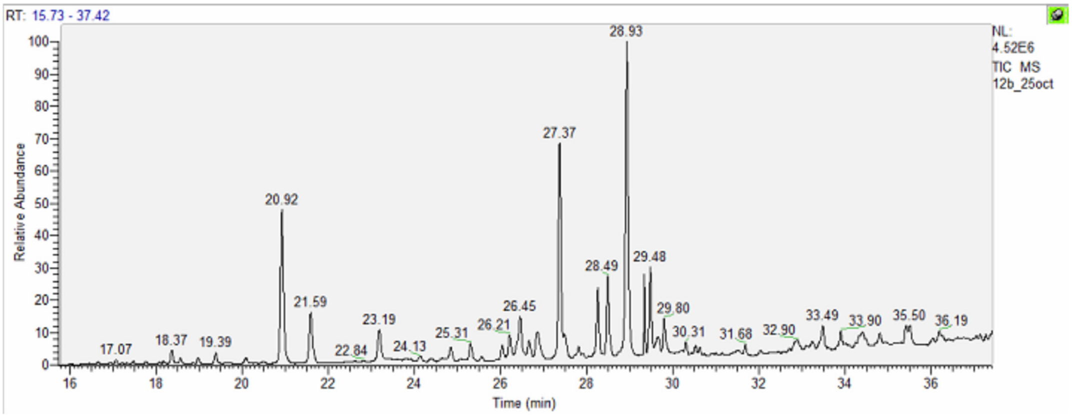

Figure S2. Gas chromatography-mass spectrometry (GC-MS) analysis of acid EPS obtained from autotrophic culture of *T. suecica.*

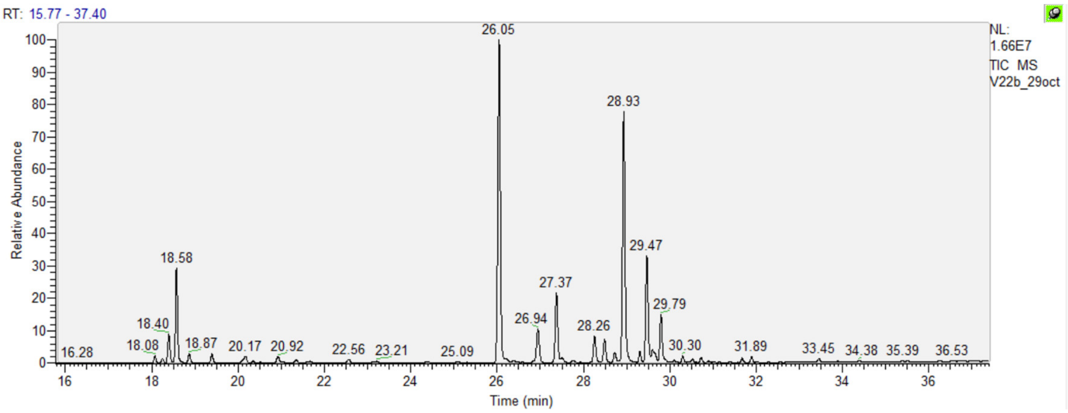

Figure S3. Gas chromatography-mass spectrometry (GC-MS) analysis of total EPS obtained from heterotrophic culture of *T. suecica.*

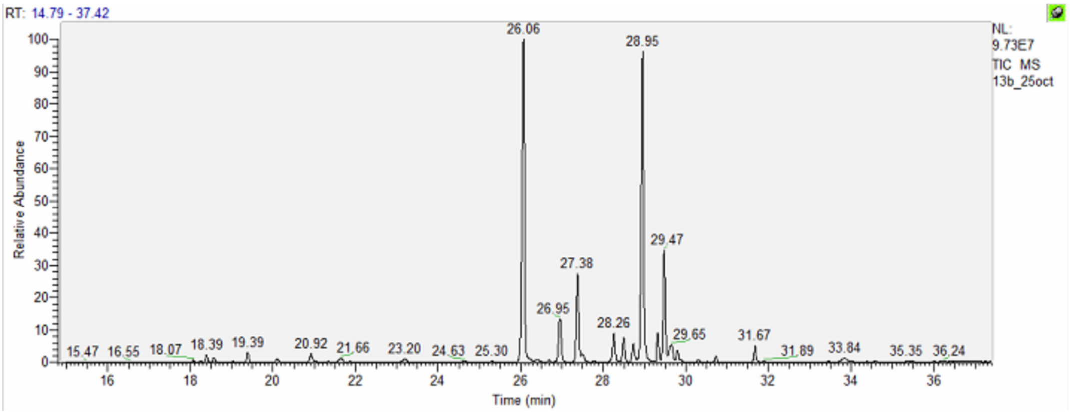

Figure S4. Gas chromatography-mass spectrometry (GC-MS) analysis of acid EPS obtained from heterotrophic culture of *T. suecica.*

© 2020 by the authors. Licensee MDPI, Basel, Switzerland. This article is an open access article distributed under the terms and conditions of the Creative Commons Attribution (CC BY) license (http://creativecommons.org/licenses/by/4.0/).
